# Molecular imaging of cellular immunotherapies in experimental and therapeutic settings

**DOI:** 10.1007/s00262-021-03073-5

**Published:** 2021-10-17

**Authors:** Nourhan Shalaby, Veronica Phyllis Dubois, John Ronald

**Affiliations:** 1 Department of Medical Biophysics, Schulich School of Medicine and Dentistry, Western University, London, Canada; 2 Robarts Research Institute, London, Ontario Canada; 3 Lawson Health Research Institute, London, Ontario Canada

**Keywords:** Immunotherapies, Molecular imaging, Cell tracking, Imaging probes, Reporter genes

## Abstract

Cell-based cancer immunotherapies are becoming a routine part of the armamentarium against cancer. While remarkable successes have been seen, including durable remissions, not all patients will benefit from these therapies and many can suffer from life-threatening side effects. These differences in efficacy and safety across patients and across tumor types (e.g., blood vs. solid), are thought to be due to differences in how well the immune cells traffic to their target tissue (e.g., tumor, lymph nodes, etc.) whilst avoiding non-target tissues. Across patient variability can also stem from whether the cells interact with (i.e., communicate with) their intended target cells (e.g., cancer cells), as well as if they proliferate and survive long enough to yield potent and long-lasting therapeutic effects. However, many cell-based therapies are monitored by relatively simple blood tests that lack any spatial information and do not reflect how many immune cells have ended up at particular tissues. The ex vivo labeling and imaging of infused therapeutic immune cells can provide a more precise and dynamic understanding of whole-body immune cell biodistribution, expansion, viability, and activation status in individual patients. In recent years numerous cellular imaging technologies have been developed that may provide this much-needed information on immune cell fate. For this review, we summarize various ex vivo labeling and imaging approaches that allow for tracking of cellular immunotherapies for cancer. Our focus is on clinical imaging modalities and summarize the progression from experimental to therapeutic settings. The imaging information provided by these technologies can potentially be used for many purposes including improved real-time understanding of therapeutic efficacy and potential side effects in individual patients after cell infusion; the ability to more readily compare new therapeutic cell designs to current designs for various parameters such as improved trafficking to target tissues and avoidance of non-target tissues; and the long-term ability to identify patient populations that are likely to be positive responders and at low-risk of side effects.

## Introduction

The immune system plays a critical role in the surveillance, progression, and regression of malignant cells throughout the lifetime of an individual. As such, cell-based cancer immunotherapies have been in development for many decades but only recently have remarkable successes been seen in otherwise treatment-refractory patients. Many immunotherapies have been, or are being developed, that are based on the isolation of particular immune cells, their ex vivo expansion, their potential priming or genetic engineering/editing, and their reinfusion back into patients. These therapies have used a variety of immune cells such as T cells, natural killer (NK) cells, and dendritic cells (DCs), amongst others. The goal is to boost a patient’s immune system to overcome immunological barriers developed by tumors to increase the capacity to kill cancer cells faster than they can grow and spread further. For an effective treatment response, it is important that the therapeutic cells are injected accurately, home to the correct locations such as to tumor(s) or lymph nodes, proliferate and/or persist long enough at these sites to invoke sufficient therapeutic effects. Currently, biopsies and blood tests are being used in the clinic to assess treatment response, however, these tests can only provide static and one-dimensional information on the total number of circulating cells or other biomarkers (e.g., cytokines). Dynamic information about whole-body immune cell responses in both tumors and normal tissues is largely lacking. As individual patient responses and treatment side effects are significant concerns with immunotherapies, we need better tools to dynamically assess immune responses to provide better patient management.

Molecular and cellular imaging tools are rapidly being developed to image cellular immunotherapies to provide non-invasive measures of cell location(s), activation, and persistence in real-time and overtime. This important information will guide clinical decisions such as optimal dose for particular patient populations, re-dose for certain individuals or termination of therapy for others, optimal routes of administration for higher on-tumor and minimal off-tumor accumulation, predictive indicators of side effects, and surrogate early measures of response/non-response. For these reasons, enormous interest has been placed on developing imaging tools for sensitive and specific in vivo monitoring of immune cells during both the preclinical evaluation and translation phases of cellular immunotherapy development going forward. Traditional strategies for non-invasively visualizing immune cells include ex vivo immune cell loading with imaging probes prior to adoptive transfer, ex vivo genetic modification of immune cells with imaging reporter genes, and the development of targeted imaging probes for in vivo labeling of immune cells. This review will focus on the first two ex vivo labeling strategies but information on the development of targeted probes for in vivo labeling and imaging of immune cells can be found elsewhere [1].

## Which imaging modality to use?

In the preclinical imaging world, high-throughput optical imaging technologies such as bioluminescence imaging and fluorescence imaging with the potential for even single-cell detection have been extensively used and have provided valuable information on our understanding of cellular response to immunotherapies in small animal models. However, these tools are limited with respect to spatial resolution, poor tissue penetration of the optical signal as well as being largely restricted from use in patients (although intraoperative fluorescence imaging is widely used and provides valuable information). Clinical imaging modalities such as positron emission tomography (PET), single-photon emission computed tomography (SPECT), magnetic resonance imaging (MRI), and photoacoustic imaging (PAI) can deploy imaging tracers, contrast agents, and/or probes to improve image contrast and have been used in both preclinical and clinical settings. Preclinical scanners that have higher spatial resolution than their clinical counterparts for each of these modalities also exist.

The primary advantages of PET and SPECT are that they are highly sensitive to low radiotracer concentrations (pmol-μmol), provide quantifiable information with unlimited penetration depth, provide high signal-to-noise ratio images, and a wide variety of clinical radiotracers are available, and more are being continually developed. Moreover, these new tracers can be rapidly translated into patients as the doses used are far below pharmacological doses. However, Krackhardt’s group has shown negative biological impacts on T cell depletion even with the use of trace doses which stresses the need for extensive functional and cytotoxic assays in the development of imaging probes for cell tracking applications [2]. Additionally, a limitation for longitudinal imaging with PET/SPECT is the use of ionizing radiation via a radioactive tracer, which also requires either a cyclotron or a generator for production in a facility of near proximity for short-lived radioisotopes. Furthermore, the spatial resolution of these systems is relatively low (4–5 mm for contemporary PET clinical scanners [3] and 1.5–2.5 mm for preclinical scanners [4]), and some tracers have high non-specific uptake, making it difficult to visualize target cells in areas of the body with high background.

In contrast to PET and SPECT, MRI uses non-ionizing radiation, can acquire high-resolution images (0.5 mm for 1.5 and 3T clinical scanners [5] and ~ 100–200 micron isotropic images for preclinical scanners) with high tissue contrast, and is generally more accessible and safer for longitudinal imaging. However, MRI suffers from low probe sensitivity (mmol range) which can make tracking low numbers of cells a challenge if ways to boost either the contrast agent dose, how much probe is in each cell, or the contrast generated per mole of agent are not explored. Importantly, although not often recognized, clinically approved MRI agents, such as gadolinium-based contrast agents (GBCA), are routinely injected at orders of magnitude higher dose compared to PET or SPECT tracers, which can help mitigate its relatively lower probe sensitivity. While several studies have reported evidence of gadolinium deposition in the brain after GBCA administration via human autopsy studies in deceased patients [6, 7], no pathological consequences have been reported and many GBCAs are still routinely used in the clinic. MRI can also image various nuclei (e.g., ^1^H and ^18^F) using the same hardware, and advances in acquisition of MR pulse sequences and hardware (radiofrequency coils and gradient coils) are constantly driving the ability to generate different contrast mechanisms that can improve the conspicuity of various tissues and cells.

Photoacoustic imaging (PAI) is a relatively newer clinical imaging modality. PAI uses non-ionizing lasers to cause thermoelastic expansion of chromophores in tissues which can then be detected externally with ultrasound transducers to reconstruct images. PAI achieves high specificity and spatial resolution, albeit within a limited field of view. The main drawback to PAI is that it has limited depth penetration (~ 5 cm) which means that one should have some prior knowledge regarding the location of the cells to be useful (e.g., after localized cell injection or known location of tumors). Despite being a relatively new field, PAI has been used in numerous preclinical models to image a variety of immune cells such as DCs, NK, and T cells.

It should be mentioned that multi-modal hybrid scanners such as SPECT/CT (computed tomography), PET/CT, and PET/MRI are also available, which can overcome limitations of individual modalities. However, the CT or MRI portion of these hybrid systems is traditionally used to provide only anatomical or functional information for the interpreting the SPECT/PET data. Molecular imaging strategies that leverage the advantages of both SPECT/PET and MRI/CT to provide complementary information regarding therapeutic immune cell fate are being developed and may help somewhat justify the relatively higher cost of PET/MRI systems.

### SPECT/PET probe labeling

#### ^111^Indium and ^99m^technetium labels

For decades, planar scintigraphy has been used to image inflammation and infection by ex vivo labeling of white blood cells (WBCs) with radiotracers such as ^111^Indium-oxine ([^111^In]In-oxine) and ^99m^Tc-hexamethyl propylene amine oxime ([^99m^Tc]TcHMPAO) [8]. Upon reinfusion into a patient, the labeled WBCs will migrate to inflamed or infected regions and can be visualized with SPECT/CT. This imaging technique provides important information on infection location and assists in optimal patient management. Particular WBCs have also been labeled with numerous radiotracers for detection in cancer patients. Matera et al. labeled activated and expanded NK cells with [^111^In]In-oxine before administration into three patients with colon carcinoma that had metastasized to the liver [9]. They were able to demonstrate homing and concentration of the NK effector cells into metastatic liver lesions with SPECT. In 2003, De Vries et al. radiolabelled DC cells with [^111^In]In-oxine, and successfully tracked DC cell migration into the lymph nodes in melanoma patients using SPECT [10]. Another group evaluated the homing ability of HER2-specific T cells in a breast cancer patient with bone metastasis using concurrent SPECT/CT and PET/CT [11]. A ^111^Indium tracer was used to radiolabel the T cells and SPECT imaging showed trafficking and infiltration to metastatic sites within 24 h post-infusion, which persisted up to 14 days. No effects on T cell viability were observed in vitro and the tracer did not diminish cytokine IFN-gamma secretion, a surrogate measure of cytotoxicity. [^111^In]In-oxine labeled gamma delta (γδ) T cells infused in patients with advanced solid tumors showed rapid migration of cells to the lungs for 4–7 h followed by a decrease in lung signal and migration of cells to the liver and spleen after 24 h [12]. Ridolfi et al. compared the migration potential of mature DCs (mDC) with immature DCs (iDC) using [^99m^Tc]TcHMPAO and [^111^In]In-oxine with SPECT imaging in 8 cancer patients (7 melanoma and 1 renal cell carcinoma). Migration of mDC to the lymph nodes was 6–eightfold higher than iDCs with initial migration occurring between 20 to 60 min post cell administration and maximum accumulation occurring between 48 and 72 h [13]. For more extensive information on [^111^In]In-oxine and [^99m^Tc]TcHMPAO cell labeling, guidelines provided by [14] and [15], respectively, should be followed.

##### ^11^Carbon labels

Although cell labeling yield is high, current SPECT probes tend to have high cellular efflux rates and result in suboptimal image quality. In some clinical scenarios, an accurate estimate of a smaller number of cells is required, which has paved the way for imaging with PET; an imaging modality that provides higher sensitivity enhanced spatial resolution as well as improved quantification. Radioactive carbon (^11^C) has been investigated as a potential tracer for PET-based cellular tracking in cancer models. Melder et al*.* used activated murine NK cells labeled with ^11^C methyl-iodide and injected these labeled cells intravenously into a fibrosarcoma-bearing C3H mouse model. Tumor radioactivity accumulation was found 30–60 min post-injection. Importantly, the authors were not only able to be used to visualize the biodistribution of NK cells, but also could quantify the number of cells within the tumor [16]. However, ^11^C, very rapidly decays with a half-life of only 20 min, making it necessary to have high starting activity to achieve synthesis, purification, and then have enough remaining activity to inject and image. Thus, this tool is primarily restricted to very short-term imaging of immune cells that will accumulate relatively quickly at their intended target tissue.

##### [^18^F]FDG labeling

Other radiotracers containing radionuclides with longer half-lives such as ^18^F, ^64^Cu, and ^89^Zr have also been explored for tracking immune cells. ^18^F-labeled fluoro-2-deoxy-2-D-glucose ([^18^F]FDG) is the most commonly used PET tracer for imaging cancer but has also been used for ex vivo labeling and tracking of therapeutic immune cells. Meier et al*.* engineered NK-92 cells to express a HER2/neu-targeting chimeric antigen receptor (CAR), radiolabelled them cells with [^18^F]FDG, and administered the cells into mice bearing HER2 + /neu breast cancer and were able to monitor the accumulation of these labeled cells in tumors [17]. Moreover, one study reported that activated human T lymphocytes radiolabeled with [^18^F]FDG showed high radiotracer efflux, as well as reduced cytotoxic and proliferative abilities [18]. Although ^18^F has a longer half-life than ^11^C, it still only allows for ~ 2–3 h of in vivo cell tracking, which may limit its appropriateness for certain applications but serves as an attractive radionuclide for repetitive imaging which also makes studying kinetics in vivo feasible.

##### ^64^Copper labels

Another radiolabel, Copper-64 (^64^Cu), with a half-life of 12.7 h, can allow for tracking of cells over a longer time period (24–36 h). In one study, ^64^Cu -pyruvaldehyde-*bis*(N^4^-methylthiosemicarbazone) ([^64^Cu]Cu-PTSM) was used to ex vivo label leukocytes for tracking in a C6 glioma rat model [19]. PET images taken up to 19 h post-infusion revealed the migration of the lymphocytes from the lungs to the spleen. Although ^64^Cu enabled longitudinal monitoring relative to ^18^F probes, ^64^Cu cell-labeling is also limited by its rapid efflux from labeled cells resulting in non-specific liver uptake.

##### ^89^Zirconium labels

More recently, ^89^Zr with a half-life of 3.2 days, has also been used to radiolabel human leukocytes and shown to have higher cellular retention than, ^111^In, ^64^Cu, and ^18^F, allowing for imaging for as long as 7-days post-injection [20]. The use of zirconium-89 has also been considerably used in immune pet to detect cell distribution with high specificity using a variety of antibodies [21, 22,23]. Weist et al. successfully imaged [^89^Zr]Zr-oxine-labelled CAR T cells with a PET/CT system in two murine xenograft models; one where mice bearing glioblastoma tumors were intracranially administered IL13Rα2-targeted CAR T cells, and the other where mice bearing PC3 prostate tumors were intravenously administered prostate stem cell antigen (PSCA) targeting CAR T cells [24]. Fruhwirth’s group was first to use [^89^Zr]Zr(oxinate)_4_ to label γδ-T cells using liposomal alendronate in a xenograft model of breast cancer to allow in vivo tracking with PET and showed increased homing of the γδ-T cells and ^89^Zr signal at tumor sites [25]. The longer half-life of ^89^Zr allowed visualization of labeled γδ-T cells over 48 h at tumor sites, with minimal effects on proliferation and cytotoxic abilities as well as DNA damage (below 20 mBq/cell) of the T cells, suggesting the benefit of using [^89^Zr]Zr(oxinate)_4_ for cell tracking. In another study, [^89^Zr]Zr-oxine was again used to label and image CAR T and NK cells in NSG mice bearing subcutaneous glioma xenografts. The authors noted the intracellular retention of [^89^Zr]Zr-oxine matched the expected in vivo lifespan of both CAR T and NK cells and can thus serve as a valuable label for tracking the biodistribution and fate of these particular cell therapies [26]. Several other studies have used ^89^Zr probes to label and track survival, proliferation and function of transgenic T cells, DC cells, NK cells, and cytotoxic T lymphocytes (CTLs) with PET [27] 20.

Importantly, there was little evidence showing that ^89^Zr probes had any negative effects on cellular viability or function, making this technique highly useful for relatively longer-term probe-based tracking of various immune cell therapies.

In vitro radiotracer labeling of cells has the most translational potential in terms of cell tracking techniques as it is fairly straightforward, sensitive (as low as 10,000 cell detection reported with PET [8]) and numerous probes for different imaging modalities have already been translated. However, this technique has issues for long-term cell tracking as the label will dilute during cell division and the imaging signal will eventually not reflect cell numbers or cells will not be detectable. This limits probe labeling to traditionally short-term studies for dividing cell populations but should be useful for non-proliferating or terminally differentiated cell types. This hurdle can be overcome by engineering immune cells with PET reporter genes that will be passed to daughter cells allowing life-long cellular imaging.

### SPECT/PET reporter genes

By far, the herpes simplex virus type 1 thymidine kinase (HSV1-tk) gene and HSV1-sr39tk, its more potent mutated derivative, have been the most used PET reporter genes [28, 29]. This viral kinase was originally developed as a suicide gene due to its ability to phosphorylate nucleoside analogs, such as acyclovir, ganciclovir, and penciclovir, administered at pharmaceutical doses. Upon entry into cells, these agents undergo phosphorylation and entrapment in HSV1-tk expressing cells with lower affinity to the human enzyme [28]. Viral thymidine kinase serves a secondary function as a PET reporter gene when administered at tracer level doses with the radiolabelled versions of these nucleosides, most commonly, 9-[4-[^18^F]fluoro-3-(hydroxymethyl)butyl]guanine ([^18^F]FHBG), 9-[(3-[^18^F]fluoro-1-hydroxy-2-propoxy)methyl]guanine ([^18^F]FHPG), [^124^I]2'-fluoro-2'-deoxy-5-iodo-1-beta-D-arabinofuranosyluracil ([^124^I]FIAU), and 2'-[^18^F]fluoro-5-ethyl-1-beta-D-arabinofuranosyluracil ([^18^F]FEAU), to allows for detection with PET [30]. Many studies have compared these analogs for visualizing HSV1-tk expression [31, 32, 33,34,35]. Amongst the Fluorine-labelled acycloguanosine, [^18^F]FHBG has high affinity for both HSV1-tk and its mutant HSV1-sr39tk [34], while [^18^F]FEAU was shown to have the highest accumulation in HSV1-tk positive cells compared to other acyclic guanosine derivatives such as [^18^F]FHPG and [^18^F]FHBG [31]. However, the Iodine-labelled [^124/125^I]FIAU was shown to have significantly higher in vitro and in vivo cellular accumulation compared to [^18^F]FHPG, [^18^F]FIAU, and [^18^F]FHBG in various HSV1-tk expressing cell lines [32, 33]. A direct comparison between [^124^I]FIAU and [^18^F]FIAU showed that [^18^F]FIAU can be used for repeat imaging with PET due to its shorter half-life while the longer half-life of [^124^I]FIAU allows visualization for several days post tracer injection [35].

The HSV1-tk reporter gene system has been in development for over 20 years, and recently the Gambhir group and colleagues were the first to report its use to track cell-based immunotherapy in patients [36]. In these studies, cytotoxic T lymphocytes (CTLs) were engineered to co-express a CAR targeting IL13Rα2, which is highly expressed in 50% of glioma patients, and HSV1-tk for PET imaging. The engineered CTLs were then infused intracranially into patients with glioblastoma. Excitingly, compared to pre-CTL images, post-CTL PET images taken after administration of [^18^F]FHBG showed significantly increased radiotracer trapping in the brain lesions, suggesting HSV1-TK-expressing CTL trapping of the tracer and longitudinal monitoring of CTLs.

A potential concern for any reporter protein not derived from humans, such as the virally-derived HSV1-tk, is the potential for it to induce an immune response and trigger the death of your chosen therapeutic cells. In fact, evidence of HSV1-tk being immunogenic in man has been reported [37]. This has led to increased interest over the years in the development of human-derived reporter genes and their application to tracking immunotherapies. Doubrovin et al. engineered T cells to express the human norepinephrine transporter (hNET), which can be imaged with SPECT or PET using a clinical-grade metaiodobenzylguanidine (MIBG) tracer radiolabelled as [^123^I]MIBG or [^124^I]MIBG, respectively (Doubrovin et al. 2007). NET-expressing T cells were administered into immunodeficient mice bearing human EBV + lymphoma xenografts expressing their restricted human leukocyte antigen (HLA) allele. T cells were visualized at tumor sites by day 1 after infusion and could be seen to continually and selectively accumulate in EBV + tumors for up to 28 days later [38].

Another human reporter gene was developed by Lee’s group; the human sodium iodide symporter (NIS) which is endogenously expressed in the thyroidal tissue, stomach, and salivary glands and functions to actively uptake iodide for hormone production. Lee’s group engineered NIS into DCs and was able to successfully monitor the migration of NIS-expressing DC cells to lymph nodes using both ^124^I^−^ as well as [^18^F]tetrafluoroborate ([^18^F]TFB) for PET/CT detection in a mouse model [39]. Emami-Shahri’s et al. has also utilized the NIS in CAR T cells with administration of [^99m^Tc]TcO_4_^−^ radiotracer for SPECT/CT [40]. Cellular detection limit of 1,000 cells using NIS with [^99m^Tc]TCO_4_^−^ using a SPECT/CT system has been shown [41]. Likewise, NIS with [^18^F]TFB revealed a cellular detection limit of 2000 cells with PET/CT [42]. NIS has also been used with pan-ErbB-targeted CAR T cells in two different triple-negative breast cancer xenograft models and reported a detection sensitivity of approximately 3000 cells with PET [43]. Like HSV1-tk, NIS also has the potential to serve as a dual reporter and therapeutic gene when administered with ^131^I, ^211^At, [^188^Re]ReO_4_^−^ and [^186^Re]ReO_4_^−^ [44, 45, 46,47, 48]. While NIS has shown higher sensitivity, contrast, and specificity compared to state-of-the-art [^18^F]FDG [42, 41], one should be aware that NIS can suffer from low target to background contrast in areas with endogenous NIS expression such as in the thyroid, salivary glands, stomach, as well as in lactating mammary glands.

The prostate-specific membrane antigen (PSMA) is a human transmembrane protein that possesses many properties that make it a desirable reporter for imaging. It has low background since it is primarily expressed in the prostate, the proximal tubules of the kidney, and the brain. Pomper’s group truncated PSMA so it would not be capable of internalizing its ligands which enabled prolonged PET probe binding and increased overall imaging sensitivity. They engineered CD19-CAR T cells to also express truncated PSMA and injected them into NSG mice with nalm6 model of acute lymphoblastic leukemia (ALL) and demonstrated that these cells can be tracked with [^18^F]DCFPyL PET. Importantly, they also showed that this technique had a detection limit of approximately 2000 cells [49]. Table 1 summarizes the studies referenced in this review with their respective modality, imaging probe, immunotherapy, targeted cell type, and tumor model.

**Table 1:** An overview of the radioisotopes used and their characteristics (half-life, emission, abundant energy, and medical use)

Radioisotope	T_1/2_	Emission	Most abundant energy (KeV)	Medical use
[^123^I]I^−^	13.2 h	gamma ray	159	Diagnostic imaging (gamma camera)
[^124^I]I^−^	4.2 days	positron/gamma ray	511/602	Diagnostic Imaging (PET)
^131^I-I^−^	8.0 days	beta ray/gamma ray	606/364	Therapy/ Diagnostic imaging (gamma camera)
[^99m^Tc]	6.0 h	gamma ray	140	Diagnostic imaging (gamma camera)
^18^F	109.8 min	positron	511	Diagnostic imaging (PET)
^89^Zr	3.3 days	positron	511	Diagnostic imaging (PET)
^111^In	2.8 days	gamma ray	171/247	Diagnostic imaging (gamma camera)
^64^Cu	12.7 h	beta ray/ positron/ gamma ray	579/656/ 511–1346	Diagnostic imaging (PET) Therapy
^11^C	20.3 min	positron/gamma ray	511/967	Diagnostic imaging (PET)
^186^Re	90.6 h	beta ray/gamma ray	1,070/59	Therapy
^188^Re	17.0 h	beta ray/gamma ray	2,120, 155	Therapy
^211^At	7.2 h	alpha ray/X-ray	7,500/77–92	Therapy

### MRI probe labeling

Iron oxide nanoparticles (IONs) are agents that cause negative contrast in MR images and have been explored for over 2 decades as tools to track cells. IONs range in size from ultrasmall superparamagnetic iron oxide nanoparticles (5 to 50 nm) to micron-sized superparamagnetic iron oxide nanoparticles (greater than 0.9 μm). For most cell types, particularly phagocytic cells, cell labeling is relatively easy and is achieved by simply co-incubating the cells with IONs. Another major advantage is that ION-based cell tracking is very sensitive, even allowing single particle or single ION-loaded cell detection in mice [50]. While generally inert, some studies have shown that even coated IONs can have slight effect on proliferation rate and can pose toxicity to certain cell types at higher concentration levels [51]. Thus, the development of high relaxivity IONs, for use at lower concentrations would be beneficial. Additionally, cell detection depends on the strength of the magnet that is used for MRI as higher field strength scanners obtain higher signal. It is also well-established that iron oxides can increase reactive oxygen species via Fenton reactions where iron oxides catalyze mitochondrial hydrogen peroxide into cytotoxic hydroxyl free radicals [52].

IONs have been used extensively to image DCs and NK cells, and a few groups have successfully ION-labeled T cells [53–58]. These studies achieved ION labeling by co-incubating the cells with IONs at concentrations between 12.5 μg/ml and 200 μg/ml for between 3 to 24 h. For instance, Su et al. aimed to track NK cells by labeling them with ferumoxytol, an FDA-approved USPIO normally used to treat anemia [53]. Mice bearing hepatocellular carcinomas received transcatheter intrahepatic artery injections or intravenous injections of labeled NK cells and images were obtained 24 and 48 h after injection with a 7T scanner. Imaging helped determine the best injection route as they showed significant signal loss in liver tumors in the transcatheter injection group but only a trend towards a loss in signal in the intravenous injection group. Tremblay et al. labeled T cells with Rhodamine-B Molday ION USPIOs and imaged them after intravenous injection into mice with cervical tumors, and the T cells could be detected as signal hypointensities up to three days post-injection in the tumors using 3T MRI [55]. Dalrup-link et al. tracked HER2-CAR-NK-92 cells labeled with an SPIO agent called Resovist in mice bearing HER2 + fibroblast tumors [56]. They determined that as few as 2.5 × 10^5^ labeled CAR-NK cells could be detected with MRI at 1.5T. Twenty-four hours after intravenous injection of 5 × 10^6^ labeled CAR-NK cells, they observed signal loss in the tumors which corresponded to CAR-NK cell trafficking. IONs have also been used in a clinical trial by De Vries et al. who tracked USPIO (Ferumoxytol) labeled patient-derived DCs injected intranodally into stage III melanoma patients [58]. Images taken with 3T MRI 2 days post-injection showed signal loss in draining lymph nodes indicating that the labeled DCs successfully migrated in the majority of patients. However, importantly they also found that three of the patients had unsuccessful injections that went into perinodular fat instead of the lymph node and DCs were not detected in these patient’s draining lymph nodes. This study showed that ION labeled DCs could be safely administered to patients and detected with clinical MRI protocols.

Perfluorocarbons (PFC) were originally developed and FDA approved in the USA as an oxygen delivery agent for patients undergoing high-risk angiography [59]. ^19^F perfluorocarbons (^19^F-PFC) have gained interest as a tracer agent for MRI as it is easily taken up by cells and can be directly visualized using ^19^F MRI. Imaging of fluorine-19 (^19^F) can be achieved on multi-nuclear MRI scanner with the use of specialized radiofrequency coils that are tuned to the ^19^F signal. In contrast to iron oxides that cause negative contrast signal voids in MR images, ^19^F PFC signal can be directly detected and overlaid onto anatomical proton MR images to visualize the signal location in the body. Advantages of using ^19^F MRI are that there is no detectable endogenous ^19^F in the body, reducing the background noise in your images. Furthermore, the ^19^F MRI signal is linear to ^19^F PFC concentration so quantifying cell numbers is relatively easy if one determines the average ^19^F PFC concentration per cell after labeling in vitro. Clinical MRI scanners have shown detection sensitivity of 10^4^–10^5^ PFC labeled cells/voxel and more sensitive cellular detection of 10^3^–10^4^ cells/voxel using high-field preclinical scanners, comparable to radionuclide methods mentioned above [60].

One commercial ^19^F PFC product called CelSense has become the standard for cell tracking with ^19^F MRI and is approved in the US. Many studies have used ^19^F PFC to successfully label and detect NK, T, and DCs in vivo using ^19^F MRI [61–65]. In these studies, immune cells are labeled by simply co-incubating them with 2.5–10 mg/ml of ^19^F PFCs (CelSense) for 16 to 24 h. These studies were carried out in a variety of cancer models and using MRI scanners with different field strengths ranging from clinical field strengths (1.5–3T) to largely pre-clinical field strengths (7T–11.7T). Kennis et al. used a 7T MRI scanner to track ^19^F PFC labeled NK cells in mice with gliomas and images revealed that ^19^F signal could be detected for at least 5 days following intracerebral NK cell injection [61]. Gonzales et al. labeled T cells with ^19^F PFC and imaged them in mice bearing melanoma tumors [63]. They determined that as few as 1.5 × 10^5^ cells could be detected per voxel using 9.4T MRI and that intravenously administered labeled T cells could be detected in the liver one day following the injection. Fink et al. imaged DCs with a clinical 3T MRI scanner and quantified the number of DCs that trafficked to the popliteal lymph node one day following a footpad injection [64]. They determined that there is a correlation between the number of cells that trafficked to the lymph node and the treatment outcome in the mice. In addition to pre-clinical studies, ^19^F PFC labeled DCs have been imaged in patients during a vaccine clinical trial by Dr. Ahrens group [65]. Five metastatic colorectal cancer patients were injected intradermally with ^19^F PFC labeled DCs and imaged 4 and 24 h after injection. The DCs were successfully detected at both timepoints and by 24 h post-injection, half of the DCs had left the injection site, highlighting the use of 19F MRI to evaluate localized injection accuracy and DC migration post-injection. As an alternative to Celsense, Hingorani et al. recently labeled CAR-T cells with in-house made transactivator of transcription (TAT) conjugated ^19^F PFC nanoemulsions [66]. TAT is a cell-penetrating peptide derived from HIV that many have explored as a means to increase cell uptake of a variety of imaging probes. Labeled CAR-T cells were locally injected into subcutaneous glioblastoma tumors in mice and images were collected 2 h post-injection with an 11.7T MRI. Image analysis revealed that almost 100% of the CAR-T cells could be detected in the tumor. Although highly promising, future work evaluating the detection limit using clinical field strengths (i.e., 1.5T or 3T) and the safety of TAT conjugated PFC will be needed before clinical translation is attempted.

Gadolinium nanoparticles are another labeling agent that has been used for MRI cell tracking. Like ^19^F, gadolinium generates positive contrast in images and is a commonly used metal that is used in the clinic to detect tumors and inflammation in patients. Currently, gadolinium nanoparticles have been used for tracking DCs and T cells in pre-clinical models. Aspord et al. labeled dendritic cells with gadolinium-based nanoparticles by co-incubating the cells with 10 mM equivalents of gadolinium for 1 h [67]. Over 98% of DCs were labeled and no effects on activation processes were observed after labeling. In-vitro functionality evaluation and staining of dead cells revealed no evidence of cytotoxicity after 24 of incubation at 10 mM with the gadolinium nanoparticle. Mice were injected intravenously or intraperitoneally with labeled DCs and imaged up to 24 h later with a 7T MRI scanner. Images showed increases in signal in the spleen of the mice in both injection groups. They predict that their methods could be used to effectively detect labeled DCs that travel to LNs in humans based on the calculated in vivo cellular sensitivity of 10^5^ cells per mm^3^.

In another study by Zhang et al., a transactivator (TAT)-conjugated ultrasmall gadolinium nanoparticle was developed to quantitatively track adoptive T cells [68]. Since T cells are harder to label due to their lack of phagocytosis and small size, the TAT peptide was shown to improve internalization of the gadolinium particle and promote cellular uptake. T cells were incubated with 500 μg of gadolinium/ml for 2 h and a 95% labeling efficiency was achieved. The probe was shown to have excellent biocompatibility with excretion via the kidney with no reported adverse effects on cell viability, in vitro cytotoxicity, or cytokine expression profiles. Mice bearing gliomas were injected intravenously with labeled T cells and positive enhancement could be visualized in the tumors at 24, 48, and 72 h post-injection with MRI. No toxicities were observed in the mice after injection, but further work will need to be done to determine if this method is appropriate for clinical translation.

### MRI reporter genes

MRI reporter genes have also been developed to enable longitudinal detection of cell populations in vivo. MRI reporter genes most often include genes for enzymes that catalyze a reaction to cause MR contrast, genes for transport proteins that cause cells to take up an imaging contrast agent, or genes that sequester endogenous compounds to produce MR contrast. While numerous MRI reporter genes have been developed in the last 2 decades, only two studies have used MRI reporter genes to track cellular immunotherapies [69]. Kim et al. used ferritin heavy chain (FTH) as a reporter gene to increase iron storage within FTH-expressing cells, which then produces negative contrast in MR images similar to ION labeled cells [70]. This group transduced DCs with the FTH reporter gene and then incubated the cells with 250 μM ferric ammonium citrate to increase iron uptake in cells. The migratory ability and proliferation of FTH expressing DCs did not change significantly compared to untransduced DCs, and FTH expression did not alter the expression of co-stimulatory molecules. Mice were injected with a total of 1 × 10^7^ FTH-DCs and images acquired 48 h later using a 9.4T MRI scanner showed signal hypointensities within the draining popliteal lymph nodes. This method has potential for longitudinal cell tracking since it employs a reporter gene, but studies need to be completed to determine if animals require continuous ferric ammonium citrate administration to allows the FTH-expressing cells detected over time. Drosophila melanogaster 2’-deoxynucleoside kinase (Dm-dnk) is the other reporter gene that has recently been used to track immunotherapy. Dm-dnk produces an enzyme that phosphorylates the substrate 2’-deoxycytidine (pyrrolo-dC) which can then be detected using chemical exchange saturation transfer (CEST) MRI. Briefly, CEST is an MRI technique that uses compounds containing protons or molecules that are selectively saturated and, after exchanging this saturation, indirectly detected through the water signal. Bar-Shir et al. recently tracked DCs 48 h after they were injected into the footpad of mice using 11.7T MRI [71]. Images showed increased CEST contrast in the popliteal lymph nodes of mice that received injections of Dm-dnk-expressing DCs compared to mice that received untransduced DCs. This method is unique as they were also able to quantify the number of cells that they detected with CEST MRI. Studies investigating whether longitudinal cell tracking is possible using Dm-dnk and pyrrolo-Dc and if engineered cells can be detected at lower, clinically relevant field strengths would be valuable in the future.

The use of the rat-derived organic anion transport polypeptide (OATP) 1A1 and human-derived OATP1B3 have also been investigated by Dr. Kevin Brindle’s group [72] as well as our group [73, 74] as MRI reporter genes due to their ability to uptake a clinical gadolinium-based contrast agent. This reporter gene system has been used for cancer cell tracking in various mouse models, and ongoing work is focused on evaluating this reporter system’s clinically relevant cell types.

### PAI probe labeling

Although PAI is not a whole-body clinical imaging tool, this imaging system can be used when localized immune cell delivery is pursued, or when a priori knowledge of the expected tracking location is available. It can also be used in concert with whole body but relatively expensive cell tracking techniques such as PET and MRI to first localize where the immune cells have accumulated, and then use PAI to track these cells longitudinally in a relatively affordable way. Indocyanine green (ICG) is a PAI dye that has a long history of safe use in the clinic for many applications beyond imaging. An advantage of ICG is that it is a near-infrared (NIR) dye, which is ideal for deeper tissue imaging. Swider et al. developed ICG-containing particles which they used to label DCs with a labeling efficiency of 33% and no significant decrease in cell viability [75]. PAI was then used to visualize intramuscularly injected DCs and no toxicity was observed one-week post-injection. Their use of a clinically relevant dye and non-invasive PAI holds promise for this technique to be translational. Further testing will be needed to determine if these ICG-loaded nanoparticles are safe for use in humans and if PAI can detect loaded DCs in human lymph nodes. NIR-797 isothiocyanate is another NIR dye that has been used for cell labeling and detection with PAI. Zheng et al. labeled T cells by incubating them with 20 μM NIR-797 dye for 30 min and then imaged the labeled T cells after injection into the footpad of mice with fibrosarcoma tumors [76]. The labeling protocol resulted in a 100% labeling efficiency without causing toxicity to T cells. PAI images taken every hour for 24 h were able to show T cell trafficking from the footpad to inguinal lymph nodes with a peak signal in the lymph nodes at 4 h. Further, they could detect intravenously administered T cells accumulate in tumors from 0 to 72 h after injection with the peak tumor signal observed at 12 h. Finally, gold nanoparticles are another labeling agent that has been used to track immune cells with PAI. Piao et al. tracked DCs stimulated with tumor specific antigens in mice with breast cancer [77]. The DCs were labeled with gold nanoparticles for 12 h with no changes in cytotoxicity, expression of important cytokines, or migratory ability. Images acquired 24 h after labeled DCs were injected into the footpad of mice showed strong signal in the axillary lymph nodes. Recently, gold nanostars (GNS), were developed and used by Bin Liu et al. to track labeled NK cells using PAI [78]. Their GNS formulation did not affect cellular activity or expression of cell surface markers, and they were able to image NK cell accumulation in mouse lung tumors 24 h after administration. They also demonstrated that GNS labeled NK cells were cytotoxic against lung cancer cells in vivo.

### PAI reporter genes

Numerous PAI reporter genes have been developed including pigment enzyme reporters, auto-fluorescent proteins, reversibly photoswitchable proteins, bacterial phytochrome photoreceptors, and chromoproteins. Human PAI reporter genes have included tyrosinase and organic anion transport polypeptide 1B3 (OATP1B3). Tyrosinase converts endogenous tyrosine into the melanin pigment which is a contrast agent for PAI due to its strong absorption properties. This system has the advantage of not needing to inject a reporter probe to generate contrast from the engineered cells; however, stable and overexpression of tyrosinase with long persistence has been shown to affect cell viability and thus ways to tightly control tyrosinase expression are critical [79]. OATP1B3 encodes a liver protein that normally is partially responsible for ICG liver clearance. Our group has recently shown that ectopic expression of OATP1B3 in cells can be coupled with systemic ICG administration as a novel PAI reporter system [80]. While no cytotoxic effects were reported with ICG administration on OATP1B3 cells at doses used in this study, further safety evaluations on the intracellular retention of ICG are needed. Additionally, the liver clearance of ICG would make it difficult to visualize OATP1B3-expressing cells that are present naturally in the liver.

This system may have advantages for translation as it uses a clinically-used dye and a human-derived reporter gene, and can also act as an MRI reporter gene with the clinical gadolinium agent Gd-EOB-DTPA. Both tyrosinase and OATP1B3/ICG reporter systems have been used to track cancer development in mouse models, but have yet to be applied to studying cancer immunotherapies [80, 81]. Future studies focusing on using PAI reporter genes for tracking immune cells could provide a low-cost and point-of-care method for visualizing cellular therapies in the clinic.

### Future outlook

**Fig. 1:**
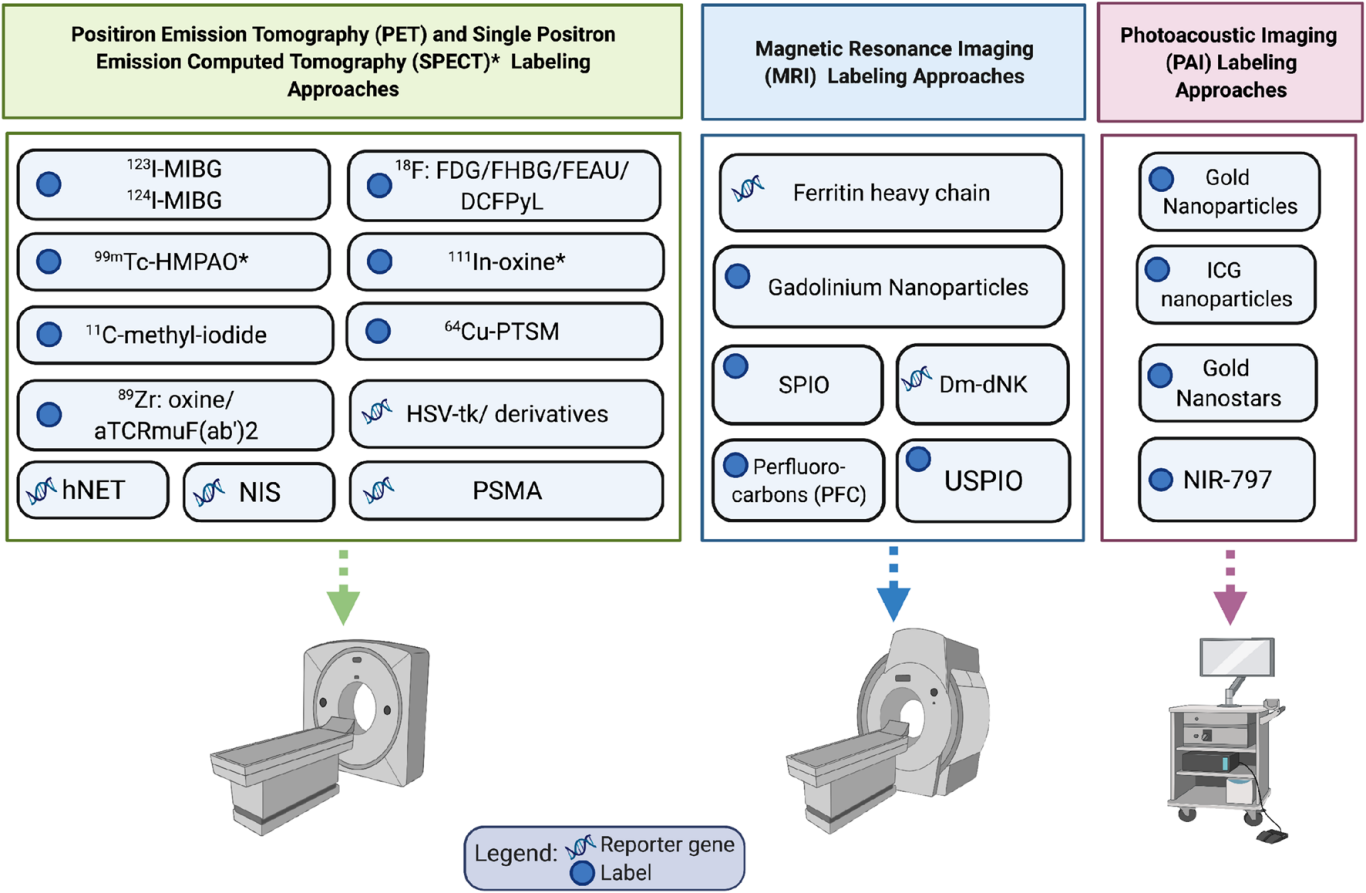
Schematic depicting the imaging modalities and labels discussed in this review. Created with BioRender.com

We would be remised if we did not talk about ultrasound imaging which also has shown utility for cell tracking. For instance, cells can be loaded with microbubbles and tracked with ultrasound [82]. Moreover, the Shapiro group has recently developed a bacterially-derived ultrasound reporter gene system that can form gas encapsulated vesicles when engineered into mammalian cells [83]. This group also utilized this acoustic reporter gene for ultrasound imaging of gene expression in mammalian cells [83]. The affordability and ubiquitous nature of ultrasound within the healthcare system make these innovative advancements very exciting. Another emerging modality that also holds promise for imaging cellular immunotherapy is magnetic particle imaging (MPI). MPI uses magnetic fields to create a field-free region that directly detects and produces hotspot images of IONs. Thus ION-based MPI is potentially advantageous over ION-based MRI cell tracking because of it pairs high sensitivity with positive contrast quantitative cell detection. Moreover, MPI images can be overlaid with CT or MR images to provide anatomical context to the ION signal. MPI was recently used to track adoptively transferred T cells using an ION called ferucarbotran [84]. Zheng, Bo et al.reported a cellular detection limit of 1000 SPIO-labelled hESCs with this preclinical modality [85]. MPI is currently limited to imaging of small animal models but a clinical MPI system is in development (Fig. 1).

Continued and early discussions and collaborations between scientists developing immune cell therapies, molecular imagers, cancer biologists, radiologists, oncologists, and regulatory agencies will be needed to accelerate the translation of “imageable” cell immunotherapy products into cancer patients. For instance, cell therapists may be reluctant to label their cells with an imaging tool due to worries about changing the behavior of the cells, or worse the efficacy of the therapy, which may elongate the process of translation. A hurdle for many reporter gene studies is ensuring that the engineering process, typically done with randomly integrating lentiviral vectors, does not impact the safety or clinical efficacy of these cellular therapies. For cellular therapies that already use viral vectors to introduce a therapeutic gene, such as CAR-T cells, this is likely to be less of a concern, but many cellular therapies do not readily engineer the cells, and scientists/clinicians may be reluctant to risk altering their long-developed therapeutic cells. Our group and others have been exploring ways to mitigate these concerns by using genome editing techniques such as zinc finger nucleases or CRISPR/Cas9 to integrate reporter genes into safe genomic harbors [86, 87]. As safer and more efficient genome editing techniques are developed, we may see more clinical studies that use reporter gene-based imaging techniques for tracking adoptively transferred cells (Table 2).

**Table 2:** Studies using molecular imaging modalities for tracking therapeutic cells

Modality	Probe or reporter gene	Labelling probe	Immune cell/target entity	Cancer cell type	Model	References
PET	Probe	[^64^Cu]Cu-PTSM	Leukocytes	C6 Glioma	Rat model	[19]
PET/SPECT	Probe	[^18^F]FDG [^111^In]In-oxine [^99m^Tc]TcHMPAO	T lymphocytes	Ovarian carcinoma cell line IGROV1		[18]
PET	Probe	[^89^Zr]Zr-aTCRmuF(ab')2	TRC T cells	Human xenograft myeloid sarcoma (ML2)	(NOD/SCID) mice	[27]
SPECT	Probe	[^111^In]In-oxine	NK (autologous primary)	Colon carcinoma	Patients	[9]
PET	Probe	[^18^F]FDG	CAR NK-92/HER2 neu NK-92-scFv(FRP5)-zeta/ HER2 neu	Breast cancer	Mice	[17]
PET	Probe	^11^C- methyl iodide	Murine NK and lymphocytes	FSaII fibrosarcoma	C3H mice	[16]
SPECT	Probe	[^99m^Tc]TcHMPAO [^111^In]In-oxine	Dendritic cells	7 melanoma and 1 renal cell carcinoma	Patients	[13]
PET	Probe	[^89^Zr]Zr-Oxine	Dendritic cells and CTLs	EL4 mouse lymphoma cells	Melanoma mouse model	[20]
PET/SPECT	Probe	^111^Indium [^18^F]FDG	CAR T cells/HER2/neu	Breast cancer	Patients	[11]
SPECT	Probe	[^111^In]In-oxine	Dendritic cells	Melanoma	Patient	[10]
PET	Probe	[^89^Zr]Zr-oxine	CAR T cells/ IL13Rα2 CAR T cells/PSCA	Glioblastoma PC3 prostate	Glioblastoma and Prostate cancer	[24]
PET	Probe	[^89^Zr]Zr(oxinate)_4_	γδ-T cells	Breast cancer xenografts	NSG mice	[25]
PET	Probe	[^89^Zr]Zr-oxine	CAR T cells NK cells	Glioma xenografts	NSG mice	[26]
PET	Reporter gene HSV-tk	[^18^F]FHBG	CAR T/ IL13Rα2	Glioblastoma	patient	[36]
PET	Reporter gene HSV-sr39 tk	[^18^F]FHBG	CAR T cells/NY-ESO-1 TCR T cells/NY-ESO-1	Jurkat cells	﻿NSG-A2.1	[29]
PET/SPECT	Reporter gene hNET	[^123^I]MIBG [^124^I]MIBG	T-lymphocytes/EBV	Lymphoma	(NOD/SCID) mice	[38]
SPECT	Reporter gene NIS	[^99m^Tc]TcO_4_^−^	CAR T cells	PC3-LN3 (PL) prostate cancer cells	(NOD/SCID) mice	[40]
PET/SPECT	Reporter gene NIS	[^18^F]TFB/ [^99m^Tc]TcO_4_^−^	Dendritic cell ﻿(DC2.4)	Imaged LNs	Mice	[39]
PET	Reporter gene PSMA	^18^F-DCFPyL	CAR T cells/CD19	Nalm6 acute lymphoblastic leukemia	NSG mice	[49]
7T MRI	Probe	USPIO	LNK cells	Hepatoma tumours	Buffalo rats	[53]
1.5T MRI	Probe	UPSIO	DCs	None	Rabbits	[54]
3T MRI	Probe	USPIO	CTLs	Cervical tumours	C57BL/6 mice	[55]
7T MRI	Probe	SPIO	BMDCs	Pancreatic ductal adenocarcinoma tumours	C57BL/6 mice	[57]
Clinical 1.5T MRI	Probe	SPIO	CAR-NK-92/HER2	HER2/neu + sarcoma tumour	BALB/c mice	[56]
MRI	Probe	SPIO	Patient derived DCs	Melanoma	Patients	[58]
19F MRI	Probe	PFC (CS-ATM-DM-green)	NK	Medulloblastoma tumour	NSG Mice	[61]
19F MRI	Probe	PFC (CS-ATM-1000)	Autologous DC vaccine	Colorectal Adenocarcinoma	Patients	[65]
19F MRI	Probe	PFC (CS-ATM-1000)	Human NK cells	Chronic myelogenous leukemia	NSG mice	[62]
19F MRI	Probe	PFC (Celsense 1000)	T cells/OVA	Melanoma	C57BL/6 mice	[63]
19F MRI (3T)	Probe	PFC (Celsense)	BMDCs	Melanoma	C57BL/6 mice	[64]
11.7T 19F MRI	Probe	PFC nanoimulsions	CAR-T cells	Glioblastoma	mice	[66]
MRI	Probe	NaGdF_4_‐TAT Nanoprobe	T cells	Glioblastoma	C57BL/6 mice	[68]
7T MRI	Probe	Gadolinium nanoparticles	Human PDCs	None	NSG mice	[67]
9.4T MRI	Reporter gene Ferritin heavy chain RG		DC2.4 cells	None	C57BL/6 mice	[70]
11.7T CEST MRI	Reporter gene Drosophila melanogaster 2′-deoxynucleoside kinase (Dm-dNK)	Pyrrolo-dC	DCs	None	C57BL6 mice	[71]
PAI	Probe	Gold nanostars	NK cells	Lung carcinoma tumors	Nude mice	[78]
PAI and 19F MRI	Probe	PFC and ICG nanoparticles	DCs	None	C57BL/6 mice	[75]
PAI	Probe	Gold nanoparticles	DC2.4 cells	Breast cancer	C57BL/6 mice	[77]
PAI	Probe	NIR‐797 label	T cells/OVA	Fibrosarcoma tumour	BALB/c nude mice	[76]

Overall, cell tracking using non-invasive imaging has been shown to provide valuable insight into the behaviors of adoptively transferred cells. Pre-clinical imaging is important to study the behavior and effectiveness of cell therapies, to compare the effectiveness of newer iterations of a cell therapy, and to optimize injection routes. Clinical implementation will enable clinicians to understand the circumstances in which treatments are effective or ineffective and the behavior of cells when side effects occur. It also may improve outcomes of clinical trials by enabling patient-specific treatment optimization. Continued development of novel labeling approaches and optimized imaging parameters that aim to improve cell detection over time will benefit cell tracking in both pre-clinical and clinical settings.

## Data Availability

N/A.
